# Lacertus fibrosus release in proximal median nerve entrapment- a systematic review

**DOI:** 10.1007/s00264-025-06493-5

**Published:** 2025-03-14

**Authors:** Qutaiba N. M. Shah Mardan, Alreem Al-khayarin, Fadi Bouri, Mohammed Muneer

**Affiliations:** 1 Hamad Medical Corporation, Doha, Qatar; 2 Qatar University, Doha, Qatar

**Keywords:** Lacertus syndrome, Lacertus fibrosus, Proximal median nerve entrapment, Scratch collapse, Lacertus notch

## Abstract

**Background:**

The role of lacertus fibrosis as the primary perpetrator behind the illusive pronator teres syndrome is becoming increasingly recognized in recent literature. The aim of this systematic review is to explore the outcomes of lacertus fibrosis release in patients complaining of proximal median nerve entrapment signs and symptoms.

**Methodology:**

In this systematic review, Pubmed, Cochrane Library, Scopus, Ovid databases were reviewed. Studies in which structures, other than the lacertus fibrosus, in the proximal forearm had been concomitantly released were deemed illegible. Various outcome assessment tools were utilized; those were pain, numbness, and satisfaction visual analog scales, return of function and muscle strength, quick DASH, work DASH, and activity DASH scores. Adherence to PRISMA guidelines was maintained.

**Results:**

A total of seven studies, three interventional and 4 retrospective observational studies, were included in this review out of 118 articles. These included 446 participants who underwent lacertus fibrosis release with a mean age of 45 years old across a mean duration of postoperative follow-up of 16.1 months. A significant proportion of the patients had a history of unsuccessful conservative or surgical management (prior carpal tunnel release in 10.5%). Minimal access surgery under WALANT was performed in 95%, US-guided release under WALANT in 3.3%, and open exploration was done in 1.5%. A horizontal incision hidden in the elbow flexion creese was done in 74.2%, oblique incision 2 cm distal and 2 cm radial to the medial epicondyle in 20.8%, and open exploration through a Z-shaped incision over the antecubital fossa in 1.5%. Immediate pain relief and return of function and strength was reported in 99.6%. A significant improvement was reported in postoperative quick DASH (mean = 24 points), work DASH (mean = 28.8 points), and activity DASH (mean = 44.8 points). Further, a significantly lower VAS score was obtained on pain, numbness, and paraesthesia scales. There were two complications, a case of postoperative haematoma and another case of surgical site infection. Seven patients complained of residual symptoms by the end of the follow up duration; carpal tunnel release was done in three and release of superficialis arcade was necessitated in four other cases.

**Conclusion:**

Lacertus syndrome can be optimally managed by surgically releasing the lacertus fibrosus. This can be done as a minimally invasive procedure under WALANT. A high index of suspicion is required when encountering patients with signs and symptoms of median nerve entrapment, specifically those who were treated unsuccessfully with the presumption of carpal tunnel syndrome.

## Introduction

The first step in effectively managing any condition is the accurate diagnosis and identification of the triggering pathology. The application of this principle has not been straightforward in treating median nerve compression due to the overlapping clinical presentation resulting from the different anatomic locations where the nerve is vulnerable to compression. Lacertus fibrosus (LF), a fibrous band distal to the elbow, has been an area of particular interest in the field of upper limb and peripheral nerve surgery. Recent studies, growing our understanding of this entity, showed how commonly lacertus fibrosus syndrome (LFS) is misdiagnosed as other median nerve entrapment neuropathies [1]. Approximately 32-49% of the patients who were misdiagnosed with carpal tunnel syndrome in some studies had their signs and symptoms originally instigated by LFS [2–4].

Repetitive pronation of the forearm catalyzes the process of compression, hence, athletes, dentists, and surgeons are amenable to develop LFS [1]. Patients often present due to loss of hand grip strength, easy hand fatigability, and forearm pain, in 95.6%, 73.3%, and 35.4%, respectively. Clinically, it can be recognized by a triad of motor weakness distal to the lacertus, tenderness over the compression site, and positive scratch collapse test. The dysfunctional muscles are the flexor carpi radialis, flexor pollicis longus, and the flexor digitorum profundus of the index finger [5]. The importance of Hagart’s triad cannot be overemphasized as electrodiagnostic studies and imaging modalities offer little value in diagnosis [1, 6]. The lacertus antagonistic test and taping are other two clinically helpful tests in establishing the diagnosis [7, 8].

The mainstay of LFS management is the surgical release of the LF; this can be performed under wide-awake local anaesthesia no-tourniquet (WALANT) [1]. Lifestyle modification plays a comforting role, especially early in the development of the disease [5]. Similarly, different injectables are used with varying outcomes. These include corticosteroids, with or without US guidance, and botulinum toxin-A [9, 10]. This article is the first systematic review that discusses LFS and its management and outcomes.

## Methodology

This systematic review was conducted with adherence to the Preferred Reporting Items for Systematic reviews and Meta-Analyses (PRISMA) guidelines [11]. Our literature review process covered PubMed, The Cochrane Library, Scopus, and Ovid from inception until May 2024 to identify studies in which solitary release of Lacertus fibrosus had been performed by entering the following keywords or Boolean string: (Lacertus fibrosus OR Lacertus fibrosus syndrome OR Lacertus syndrome OR bicipital aponeurosis) AND (Release OR decompression) AND (Median nerve OR median neuropathy). Two independent reviewers (Q. SM. and A. A.) evaluated the inclusion and exclusion criteria in adherence to PRISMA guidelines. In case of doubt, a third reviewer was included to discuss a consensus (M. M.).

The inclusion criteria were studies in which LF alone was released for proximal median nerve entrapment (PMNE) symptoms leaving the other structures in the elbow intact. Case series and retrospective studies were deemed eligible. We excluded the studies in which concomitant release of structures other than LF was done to treat PMNE, cadaveric, animal, studies without full-text access, book chapters, case reports, commentary letters, and articles in languages other than English. After thorough paper review, two included studies were eventually excluded due to involvement of additional decompression of structures other than LF.

The data was exported to Rayyan where screening for duplicates was done. The remaining articles were screened individually for eligibility based on the title and abstract. Next, data extraction then quality assessment was done following The MethodologicAl STandards for Epidemiological Research (MASTER) scale for risk of bias assessment [12]. The assessment was performed by two researchers (Q. SM. and A. A.) and a third researcher was consulted in case of disagreement (M. M.).

## Results

**Fig. 1:**
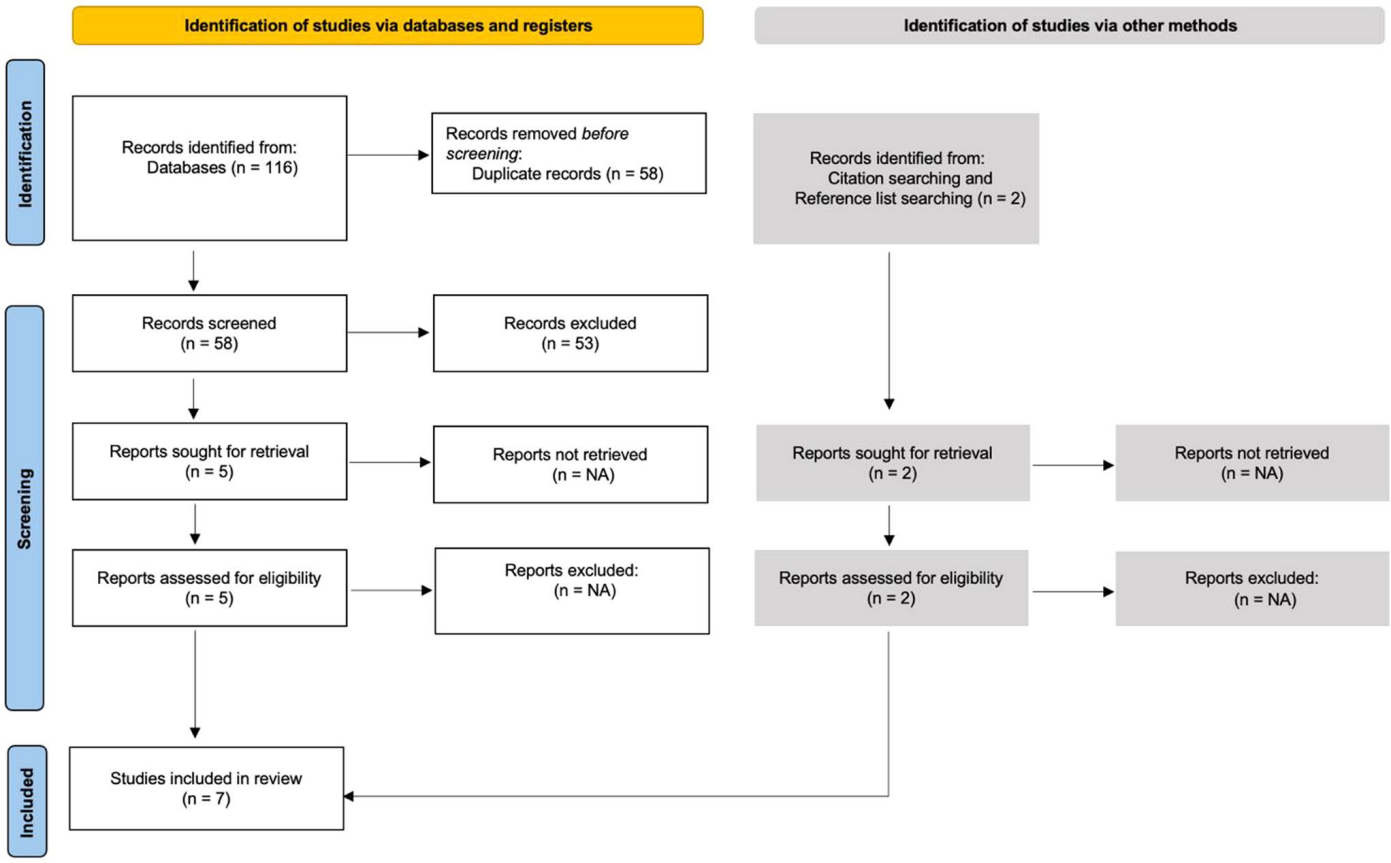
.

A total of 446 subjects were included in this study, reported in seven out of 118 papers that fulfilled the inclusion criteria; three were interventional studies and the remaining four were retrospective in nature. Please review Fig. 1 which shows the PRISMA chart flow and summarizes the selection process and Table 1 for risk of bias assessment. The mean age of the participants was 45 years old with a mean follow-up duration of 16.1 months. Four studies reported dominance (331/446 patients, 74.2%); in these, 237 patients (71.6%) complained of the symptoms in their dominant limb compared to the remaining 92 (27.7%) and two (0.6%, who complained of bilateral involvement). The remaining three studies did not report this information. Table 2 highlights the most important characteristics of the studies included. Majority of the patients received prior conservative therapy in terms of oral and injectable anti-inflammatory drugs and occupational therapy sessions; 47 patients (10.5%) were managed as cases of carpal tunnel syndrome and underwent surgical release.

**Table 1:** Risk of bias assessment using the MASTER scale

Safeguard item	Ahmed, 2023	Cline, 2023	Ayhan, 2023	Hagert, 2023	Apard, 2022	Hagert, 2013	Seitz. 2007
Data collected after the start of the study was not used to exclude participants or to select them into the analysis	1	0	1	1	1	1	1
Participants in all comparison groups met the same eligibility requirements and were from the same population and timeframe	0	0	0	0	0	0	0
Determination of eligibility and assignment to treatment group/ exposure strategy were synchronized	0	0	0	0	0	0	0
None of the eligibility criteria were common effects of exposure and outcome	1	1	1	1	1	1	1
Any attrition (or exclusions after entry) was less than 20% of total participant numbers	0	0	0	0	1	0	1
Missing data was less than 20%	1	0	1	0	1	1	1
Analysis accounted for missing data	0	0	0	0	0	0	0
Exposure variations / treatment deviations were less than 20%	1	1	1	1	1	1	1
Variations in exposure or withdrawals after start of the study were addressed by the analysis	0	0	0	0	0	0	0
Procedures for data collection of covariates were reliable and the same for all participants	1	1	1	1	1	1	1
The outcome was objective and/ or reliably measured	1	1	1	1	1	1	1
Exposures/ interventions were objectively and/ or reliably measured	0	0	0	0	0	0	0
Outcome assessor(s) were blinded	0	0	0	0	0	0	0
Participants were blinded	0	0	0	0	0	0	0
Caregivers were blinded	0	0	0	0	0	0	0
Analyst(s) were blinded	0	0	0	0	0	0	0
Care was delivered equally to all participants	1	1	1	1	1	1	1
Cointerventions that could impact the outcome were comparable between groups or avoided	1	1	1	1	1	1	1
Control and active interventions/ exposures were sufficiently distinct	0	0	0	0	0	0	0
Exposure/intervention definition was consistently applied to all participants	1	1	1	1	1	1	1
Outcome definition was consistently applied to all participants	1	1	1	1	1	1	1
The time period between exposure and outcome was similar across patients and between groups or the analyses adjusted for different lengths of follow-up of patients	0	0	0	0	0	0	0
Design and/or analysis strategies were in place that addressed potential confounding	0	0	0	0	0	0	0
Key confounders addressed through design or analysis were not common effects of exposure and outcome	0	0	0	0	0	0	0
Key baseline characteristics / prognostic indicators for the study were comparable across groups	0	0	0	0	0	0	0
Participants were randomly allocated to groups with an adequate randomization process	0	0	0	0	0	0	0
Allocation procedure was adequately concealed	0	0	0	0	0	0	0
Conflict of interests were declared and absent	1	1	1	1	1	1	1
Analytic method was justified by study design or data requirements	1	1	1	1	1	1	1
Computation errors or contradictions were absent	1	1	1	1	1	1	1
There was no discernible data dredging or selective reporting of the outcomes	1	1	1	1	1	1	1
All subjects were selected prior to intervention/ exposure and evaluated prospectively	0	0	0	0	1	1	0
Carry-over or refractory effects were avoided or considered in the design of the study or were not relevant	1	1	1	1	1	1	1
The intervention/ exposure period was long enough to have influenced the study outcome	1	1	1	1	1	1	1
Dose of intervention/ exposure was sufficient to influence the outcome	0	0	0	0	0	0	0
Length of follow-up was not too long or too short in relation to the outcome assessment	1	1	1	1	1	1	1
Summary count of safeguard out of 36	17/36	16/36	16/36	16/36	19/36	18/36	17/36

Physical examination findings and tests varied across the included studies with some similarities. Seitz et al. (2007), whose cohort was comprised of patients presenting with acute symptoms, reported that their patients held their arms in a flex, supinated, and cradled position, with a shock-like pain once flexion or supination against resistance or passive elbow extension or forearm pronation was attempted. Also, they reported tenderness over the median nerve at the antecubital fossa coexisting with a positive Tinel’s sign, weakness in grip and elbow flexion, but no sensory or motor deficit of the median nerve [13]. Hagert (2013), Apard et al. (2022), Ayhan et al. (2023) and Hagert et al. (2023) followed the clinical triad of: Weakness of the FPL, FCR, FDP-II, tenderness or positive Tinel’s sign over the LF, and positive scratch collapse test to establish the diagnosis [14–16]. Cline et al. (2023) followed a slightly different triad: Paraesthesia across the median nerve territory with hand or forearm weakness, reproduction of the pain, weakness, or paraesthesia with compression over the median nerve at the antecubital fossa, and weakness of the FPL, FCR, and FDP-II [17]. In addition to the tenderness point and weakness of the FPL, FCR, FDP-II, Ahmed et al. (2023) added paraesthesia over the thenar eminence and pain upon resisted elbow flexion in 90 degrees full supination. The latter two additions were not unanimously found in all patients, in contrast to the former two findings [6].

The included studies described three methods of LF release. Most of the patients, 95%, underwent LF release through wide-awake local anaesthesia no tourniquet protocol (WALANT) and minimal access. Microinvasive percutaneous US-guided release under WALANT was done in 3.3%; and only 1.5% underwent open exploration. Access was through a single horizontal incision in the elbow flexion crease in 74.2%, single oblique incision 2 cm distal and 2 cm radial to the medial epicondyle in 20.8%, and the open exploration was through a Z-shaped longitudinal incision over the antecubital fossa in 1.5%.

Overall, the eligible studies reported favourable outcomes. Following LF release, pain was relieved, hand function and grip strength were restored, and annoying symptoms, such as paraesthesia, improved. Different scales of outcome assessment like visual analogue scores (VAS), disabilities of the arm, shoulder and hand (DASH), patient-rated elbow evaluation (PREE), and extent of power (reported in kilogram) were utilized. A significant improvement was reported in postoperative quick DASH (mean = 24 points), work DASH (mean = 28.8 points), and activity DASH (mean = 44.8 points). Further, a significantly lower VAS score was obtained on pain, numbness, and paraesthesia scales. Details of the outcomes pertinent to each study can be found in Table 3. Notable, three cases had to undergo carpal tunnel release after LF release, two of which were revision cases [17]. Four other patients were found to have residual median nerve compression symptoms that was caused by the superficialis arcade necessitating surgical release [5]. In terms of complications, a case of postoperative haematoma [15] and another case of surgical site infection [17] were reported, both of which were managed conservatively.

**Table 2:** This table presents the demographic and general data of the eligible population

Article	Design	Population	Age	Gender	Affected limb	Prior treatment
Seitz 2007	Case series	7	Mean = 37.7 min = 16 max = 67	M = 5 F = 2	-	Oral and injectable anti-inflammatory drugs
Hagert 2013	Interventional	44	Mean = 48.8 min = 25 max = 66	M = 22 F = 22	Right = 23 (52.2%) Left = 21 (47.7%)/ Dominant = 25 (56%)	-
Apard 2022	Interventional	15	-	-	-	Conservative management
Ahmed 2023	Retrospective cohort	93	Mean = 38.7	-	-	Occupational therapy
Cline 2023	Interventional	10	Mean = 53 min = 32 max = 80	M = 4 F = 6	Dominant = 5 (50%) Non-dominant = 3 (30%) Bilateral = 2 (20%)	10 ipsilateral limbs had prior carpal tunnel release
Ayhan 2023	Case series	32	Mean = 45.1 min = 24 max = 63	M = 0 F = 32	Dominant = 23 (71.8%) Non-dominant = 9 (28.1%)	11 patients had prior carpal tunnel release
Hagert 2023	Retrospective cohort	245	Mean = 47 min = 19 max = 73	M = 138 F = 137	Dominant = 184 (66.9%) Non-dominant = 91 (33.1%)	26 patients had prior carpal tunnel release

Please note that the dashed boxes mean that the information was not reported

**Table 3:** This table details the intervention, type of surgical incision, and the postoperative results following lacertus fibrosus release

Article	Intervention	Type of incision	Follow-up	Results
Seitz 2007	Surgical exploration, division and resection of the LF	Z-shaped longitudinal over the antecubital fossa	15–48 months	Immediate pain relief and return to function, normal grip strength
Hagert 2013	LF surgical release under WALANT	Transverse, medial elbow flexion creese	6 months*	Quick DASH improvement = 22.7, work DASH improvement = 31.8, activity DASH improvement = 55.3, numbness VAS score = 1.3, pain VAS score = 0.9, satisfaction VAS score = 8.8, Immediate return of strength
Apard 2022	microinvasive percutaneous ultrasound-guided release of the LF under WALANT	-	1 month	Mean pain VAS score improvement = 5.6, regain of muscle strength and function
Ahmed 2023	LF surgical release under WALANT	Oblique, 2 cm distal and 2 cm radial to the medial epicondyle	6 months	Quick DASH improvement = 42.4, mean grip strength improvement = 8 kg, pinch strength improvement = 4 kg**
Cline 2023	LF surgical release under WALANT	Transverse, medial elbow flexion creese	60 months***	Regain of muscle strength, paresthesia VAS = 0.8, limb pain VAS = 0.8, outcome satisfaction VAS = 9.6, procedure recommendation VAS = 9.8, quick DASH = 9.1, PREE = 4.1
Ayhan 2023	LF surgical release under WALANT	Transverse, medial elbow flexion creese	1.3 month	Median satisfaction VAS = 10, tip-to-tip pinch mean improvement = 1.1 kg, lateral pinch mean improvement = 1.3 kg, tripod pinch mean improvement = 1.7 kg, mean DASH score improvement = 33.9, median improvement of VAS score pain = 8, median improvement of VAS score paresthesia = 8.5
Hagert 2023	LF surgical release under WALANT	Transverse, medial elbow flexion creese	6–9 months	Immediate return of strength in 99.2%, mean quick DASH improvement = 22, mean work DASH improvement = 25.8, mean activity DASH improvement = 34.3, pain VAS score = 1.9, numbness VAS = 1.8, satisfaction with the surgical outcome VAS = 8.5****

Please note that the dashed boxes mean that the information was not reported

* Only 71.1% of the patients in this study completed the six months follow-up

** Quick DASH scores were reported at six months follow-up, while the strength tests findings were recorded immediately postoperatively

*** There were ten patients and 12 limbs; only seven patients (Eight limbs) completed the whole 5-year-follow-up duration

**** In this study, 74.5% had isolated LF release, 25.1% had concomitant LF and carpal tunnel release, and a single patient had concomitant LF and superficialis arcade release

Abbreviations: LF: Lacertus fibrosus; WALANT: Wide-awake local anesthesia no-tourniquet; DASH: Disabilities of the arm, shoulder and hand; VAS: Visual analogue score; PREE: Patient-rated elbow evaluation

## Discussion

Along its course through the distal arm and proximal forearm, the median nerve can be compressed by the following structures [18]: Struther’s ligament [19], the LF [20], between the two bellies of the pronator teres [21], the arcade of the flexor digitorum superficialis [22], and accessory muscles [23]. Traditionally, LFS was included under the wider category of pronator teres syndrome- attributing to improper management [1]. The LF is an aponeurotic structure that emerges from the border of the distal tendon of the biceps muscle; and is in direct contact with the median nerve in over half of the individuals as it courses medially and distally down the forearm [24]. The compression at this site manifests as weakness of the flexor pollicis longus, flexor digitorum profundus of the index finger, and flexor carpi radialis [2, 14]. The details of differentiating LF from other closely resembling entities are beyond the scope of this review. The Hagert triad has been consistent in strongly suggesting the diagnosis; other complimentary tests include reproduction of the symptoms with compression over the LF, pain with resisted elbow flexion in 90 degrees full supination, paraesthesia over the thenar eminence [6, 9, 14–17]. Martinel and Apard (2024) had recently introduced the lacertus antagonistic test (LAT) but further studies are required to establish the specificity and sensitivity of the test [7]. LAT findings could be reproduced through taping, another clinical test. Finally, the presence of the lacertus notch, a contour deformity in the proximal forearm, was found in 65.1% of the forearms affected by LFS [25].

LFS has an affinity towards the dominant hand, evident by its involvement in 71.6% of the pooled sample compared to the remaining 27.7% and 0.6% who complained of symptoms in their non-dominant hand or both, respectively. This reiterates the dynamic nature of the compression neuropathy in this syndrome [24]. Moreover, the limitation in electrodiagnostic studies could be traced back to this reason as they often yield misleading results [1]. This, however, draws more attention to the role of ultrasound, as it enables real-time insight into the nerve and detects any dynamic compression process. Also, compression against the US probe while the forearm is in pronation against resistance helps in exploring the status of the median nerve [1, 26]. Most of the included studies described their surgical approach through a short horizontal incision at the elbow flexion crease, the LF band can be readily identified and released under WALANT. This method allows immediate assessment of the return of function and directs further release if required. It also negates the requirement for regional anesthesia or tourniquet application and saves on expenses [4, 5, 14, 16, 17]. Ahmed et al. (2023) used a short single incision but slightly different in that it is oblique in orientation towards the radial styloid. The incision is made 2 cm distally and 2 cm radially from the medial epicondyle. No details as for why this preference over the transverse incision in the elbow flexion crease is adapted was given, but they commented that this incision is adequate for LF release [6]. Apard et al. (2022) described a microinvasive method of releasing LF percutaneously under sonographic guidance in 15 patients [15]. It showed immediate return of power in all patients and a significant reduction in VAS for pain (From 6.2 to 0.6, *p* < 0.001). Their method can be applied in office-setting with minimal morbidity to the patient but requires expertise with operating sonography machines. Moreover, it offers visibility and clear anatomy to the operating surgeon. Open exploration and LF release through a z-shaped longitudinal incision across the antecubital fossa was described in the case series by Seitz et al. (2007). Their subjects differed from the patients in the other studies in the nature and onset of the disease; patients complained of acute symptoms following forceful elbow flexion against resistance, partially tearing the biceps brachii myotendinous junction and creating a choke point over the median nerve by the tethered LF [13]. The authors described that no other pathology was noticed intraoperatively in all their patients, which raises the possibility of managing acute LFS through minimal access without the need for the extensive exploration. In terms of complications, a case of surgical site infection treated conservatively was reported by Cline et al. (2023) and a case of haematoma that was self-remitting was reported by Apard et al. (2022) [15, 17]. Seven patients complained of residual symptoms by the end of the follow up duration; carpal tunnel release was done in three patients [17] and release of superficialis arcade was necessitated in the four other cases [5]. No recurrence was reported in all the included studies by the end of their follow-up duration.

Careful assessment of patients with features suggestive of median nerve compression is required, as Kong et al. (2023) reported double crush phenomenon in 78% of the limbs affected by median neuropathy attributed to carpal tunnel and LF. Notably, isolated carpal tunnel syndrome and LFS were observed in only 17% and 5% of the affected extremities, respectively [3]. The role of US is currently limited to ruling out solid or cystic masses or identifying static nerve compression [8]. Future studies looking into its capability at diagnosing LFS will add to the diagnostic armamentarium. The limited number of the patients and studies investigating the topic may chip away from the strength of the findings in this paper, however a consensus in the results should prompt hand and peripheral nerve surgeons into the possibility of LFS. Further, future studies should add to the body of evidence with more patients and universal measure of reporting outcomes. Also, future studies should explore the most common presentations in LFS, as the presentation is highly variably and illusive.

## Conclusion

Release of the compression exerted over the proximal median nerve by the LF results in the resolution of the weakness, fatiguability, and pain. After thorough clinical assessment and accurate diagnosis, the surgical release can be best done through a small 2-cm incision in the proximal forearm under WALANT protocol, with the results of a successful release manifesting as immediate return of power and function. Percutaneous release under ultrasound guidance is a promising modality of treatment.

## Data Availability

No datasets were generated or analysed during the current study.
